# Impact of the COVID-19 pandemic on work routine, practice and mental state of radiation oncologists in India: an online survey

**DOI:** 10.3332/ecancer.2021.1165

**Published:** 2021-01-07

**Authors:** Vinodh Kumar Selvaraja, Deleep Kumar Gudipudib

**Affiliations:** Department of Radiation Oncology, Basavatarakam Indo-American Cancer Hospital and Research Institute, Road number 10, Banjara Hills, Hyderabad 500034, Telangana, India; a https://orcid.org/0000-0002-2507-8328; b https://orcid.org/0000-0003-3447-4725

**Keywords:** COVID-19 pandemic, India, mental state, radiotherapy practice, radiation oncologist

## Abstract

**Background and purpose:**

COVID-19 has affected the lives of every medical professional including oncologists. The goal of this survey was to evaluate the impact of COVID-19 on the work routine, psychological state and radiotherapy practice of radiation oncologists.

**Materials and methods:**

An anonymous survey consisting of 23 questions regarding the lives of radiation oncologists during the COVID-19 pandemic was distributed online via social media from July 14 to July 21, 2020. Statistical analysis was performed with Statistical Package for the Social Sciences 18.0 software and basic descriptive statistics were applied.

**Results:**

A total of 82 radiation oncologists responded to the survey. The majority were professors (28/82; 34.1%) and residents (28/82; 34.1%) and <50 years old (70/82; 85.4%). Cancer screening programs (57/62; 91.9%) and number of new cases reduced (44/82; 53.7%) in many institutes. Follow-up was still done in-person by 73.2% respondents. 35/82 (42.7%) respondents were satisfied about their safety during COVID-19, at the same time 36/82 (43.9%) were worried about the patient’s safety. The fear of contracting COVID-19 (57/82; 69.5%) and infecting their families (64/82; 78%) was high. Physical presence during case implementation (59/82; 72%) and daily setup verification (60/82; 73.2%) remained the same during COVID-19. Half of the respondents adopted new fractionation schedules, commonly in breast and palliative cases. Time spent on research had reduced by 62.2%. Only 41.4% respondents were satisfied with the patient care provided by them during the COVID-19 pandemic.

**Conclusion:**

COVID-19 has significantly altered the work routine, radiotherapy practice and mental state of radiation oncologists.

## Introduction

The COVID-19 pandemic has significantly affected healthcare all over the world [1, 2]. On 30 January 2020, the first case of COVID-19 was confirmed in India. Since then, there has been a rapid spread of the virus all throughout the country [3]. As on 6 August 2020, India has reported 1,964,536 COVID-19 cases, of which 595,501 (30.31%) were active cases, 1,328,336 (67.62%)

were discharged and 40,699 (2.07%) died [4]. To tackle the ongoing COVID-19 situation, majority of the healthcare resources were reallocated for deduction and treatment of COVID-19. However, oncology services have continued amidst all these problems. Cancer societies around the world have suggested various strategies/guidelines to ensure safe delivery of cancer care during the COVID-19 pandemic [5–7]. These guidelines are continuously evolving as per the changes in the native COVID-19 situation.

In radiation oncology, various recommendations such as switching to hypofractionation regimens, definitive chemoradiation approaches and teleconsultations were put forth [8–12]. However, there is extremely limited information regarding adaptation of these guidelines by radiation oncologists, and the changes in their work routine, psychological state, radiotherapy practice and research. Thus, the primary aim of this online survey is to know the impact of COVID-19 on all of the above aspects among radiation oncologists in India.

## Materials and methods

A survey consisting of 23 questions (Online Supplementary Material—Life of Radiation Oncologists during COVID-19—Questionnaire) was created using a freely available online survey tool. The survey was structured to capture general anonymous data of respondents, data on cancer screening programmes, number of new cancer cases, follow-up of treated patients and data regarding changes in workplace during the COVID-19 pandemic. Data on safety of healthcare workers and patients, and fear of contracting COVID-19 or infecting their family members were collected. Data on changes in radiotherapy practice in terms of physical presence during new case implementation, daily setup image verification, deferral/postponement of radiotherapy due to COVID-19, adaptation of new fractionation schedules, desire to continue the new fractionation schedules post-COVID-19 and use of single fraction palliative radiotherapy for pain palliation were collected. Furthermore, data on time spent for cancer research, and professional satisfaction on patient care during COVID-19 were also collected.

The questions were single-answer multiple-choice questions to be answered by all respondents mandatorily except for one question (question 19), which was open ended and to be answered only if applicable to them. The multiple-choice questions were either based on Likert scale or polar type. Questions were primarily focused on areas where there is limited information regarding impact of COVID-19 on radiation oncologists. The survey was not formally validated or pilot tested. It was shared via social media among radiation oncologists in India, predominantly Southern India. The sample size was not pre-determined. The survey was kept open online for a period of 10 days and responses were recorded with implied consent.

Descriptive statistics such as frequency and percentage were used to analyse the results of this study. The percentages were counted from those who answered a certain question and not from the entire cohort. Whenever appropriate, the Fisher exact test or χ2 test was used to compare respondents. *p*-value of <0.05 was statistically significant. Statistical Package for the Social Sciences 18.0 software was used to perform these statistical analyses.

## Results

The survey was completed by 82 respondents and the response rate was 65%. Among the total respondents, 34.1% (28/82) were radiation oncology professors/heads of the department, 34.1% (28/82) were post-graduates, 30.5% (25/82) were assistant professors/senior consultants and 20.7% (17/82) were senior residents/registrars. A total of 85.4% (70/82) of respondents were ≤50 years old. Male and female respondents were 56.1% (46/82) and 43.9% (36/82), respectively. Respondents were from academic institutes (42/82; 51.2%), multi-specialty hospitals (24/82; 29.3%), government hospitals (10/82; 12.2%) and cancer clinics (6/82; 7.3%).

### Changes in the workplace

**Figure 1.**
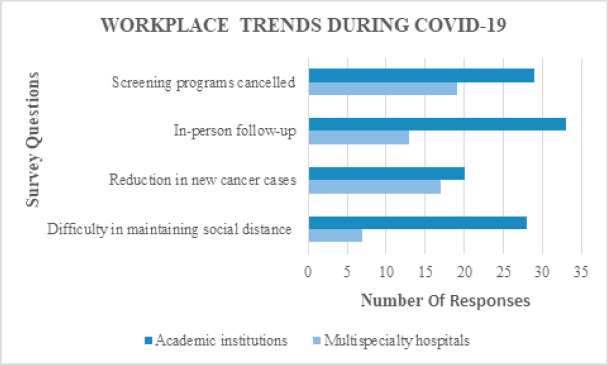
Comparison between responses of the participants belonging to academic institutions and multi-specialty hospitals regarding changes in workplace during COVID-19.

COVID-19 has affected cancer screening programmes in 91.9% (57/62) of institutions/hospitals. In almost three-fourths (60/82; 73.2%) of the institutes/hospitals, follow-up patients were examined in-person and only <15% (12/82) resorted to online/telephone consultations. The number of new cancer cases was reduced in 53.7% (44/82), while the number remained the same in 35.4% (29/82). On analysis, the decrease in number of new cancer cases was statistically significant in academic institutes and multi-specialty hospitals (*p* = 0.0484) (Figure 1). Almost half of the respondents worked full-time (39/82; 47.6%), one-third worked for reduced hours (26/82; 31.7%) and one-fifth had 3-day/weekly shifts (17/82; 20.7%). On comparing work timings of respondents based on age, respondents above 50 years had reduced work hours, which was statistically significant (*p* = 0.0279). Responses to question on maintenance of social distancing in out-patient department (OPD) were equally distributed; 48.8% of respondents were able to maintain social distance, while 47.6% found it difficult. Compared to respondents from multi-specialty hospitals, respondents from academic institutes found it more difficult to maintain social distancing in OPD (*p* = 0.0093) (Figure 1).

### Changes in psychological state

**Figure 2.**
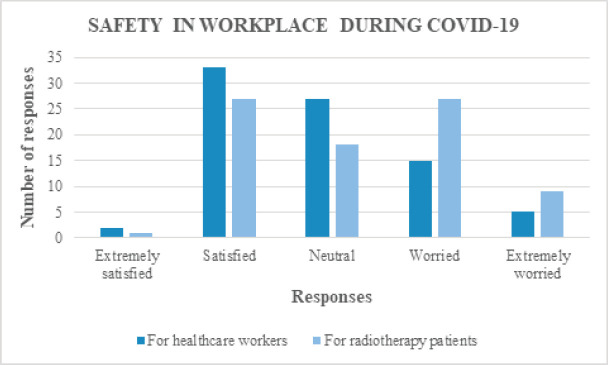
Responses for questions on safety of healthcare workers and radiotherapy patients in workplace during COVID-19.

**Figure 3.**
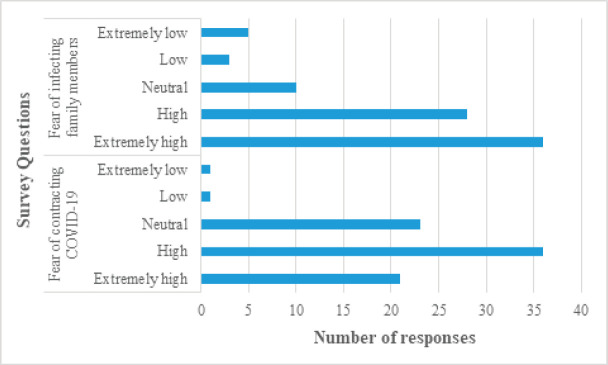
Responses of radiation oncologists regarding fear of contracting COVID-19 and infecting their family members.

For the questions on the safety of healthcare workers, 42.7% (35/82) were satisfied, 32.9% (27/82) were neutral, while 24.4% (20/82) were worried. On the other hand, regarding the safety of radiotherapy patients, 43.9% (36/82) were worried, 22% (18/82) were neutral and 34.1% (28/82) were satisfied (Figure 2). Fear of contracting COVID-19 was high among the respondents (57/82; 69.5%), of which the fear was extremely high in 36.8% (21/57). Similarly, fear of infecting family members with COVID-19 was also high (64/82; 78%) and 56.3% (36/64) had extreme fear (Figure 3). Among the respondents, 41.4% were satisfied with oncological care provided to the patients during COVID-19. Of the remaining two-thirds, almost one-third were neutral (23/82; 28%) and one-third were not satisfied (24/82; 29.3%).

### Changes in radiation oncology practice

**Figure 4.**
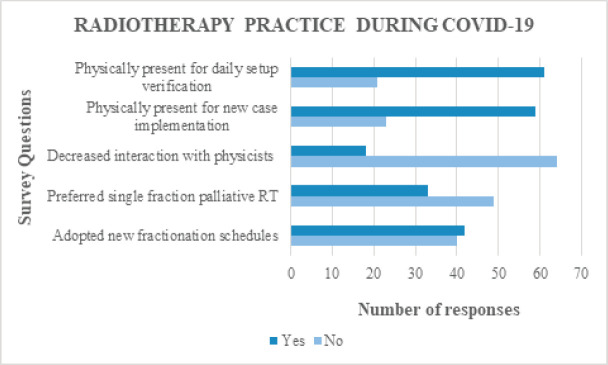
Responses of participants regarding changes in radiotherapy practice during COVID-19.

In the majority of respondents, interaction with physicists remained the same during COVID-19 (62/82; 75.6%), while it was decreased in only 22% (18/82). Almost three-fourths of the respondents were physically present during new case implementation (59/82; 72%) and daily setup image verification (60/82; 73.2%) (Figure 4). Only one-fourth reported reduced physical presence. Radiation treatment for patients was deferred in some instances by more than one-third (30/82; 36.6%) of respondents due to the COVID-19 situation. The data on adoption of new radiotherapy fractionation schedules during COVID-19 as advised by oncology societies were almost equally distributed. Among those who adopted newer fractionations (42/82; 51.2%), the common disease sites where they were employed was breast cancer (21 responses), head and neck cancer (10 responses) and palliative setting (8 responses). More than one-third wanted to continue the new radiotherapy fractionation schedules even after COVID-19 pandemic. Increased preference for single fraction palliative radiotherapy for painful bone metastasis during COVID-19 was reported by only 40.2% (33/82) of respondents (Figure 4). The time spent on cancer research and publications was reduced in the majority of the respondents (51/82; 62.2%) and was increased in only a meagre 13.4% (11/82) (Figure 4). On further analysis, 21 out of 28 postgraduates (75%) reported decreased research activities during COVID-19.

## Discussion

The results of this survey offer three primary sources of information regarding the impact of COVID-19 on workplaces, psychological state of radiation oncologists and radiation oncology practice. The COVID-19 pandemic has changed the treatment approaches in various cancer subsites and will probably continue to change in the forthcoming months too. This survey captured information from radiation oncologists across different states during the peak of COVID-19 in India. The majority of the respondents (80.5%) worked in an academic institute/multi-specialty hospital, hence wherever appropriate responses were compared between academic institutes and multi-specialty hospitals (Figure 1). A total of 91.9% of respondents reported that COVID-19 affected cancer screening programmes in their institution/hospital. In India, cancer screening programmes are still in the developing phase with only a few institutes conducting screening camps, predominantly for early detection of cervical and breast cancers [13, 14]. Therefore, the majority of the cancer patients present with bulky mass and advanced stage disease. The decrease in cancer screening camps during COVID-19 may lead to a further increase in the number of advanced cases and negatively impact the prognosis in these patients. Follow-up of cancer patients was done in-person by 73.2% of respondents and only 14.6% utilised technology to assess these patients. To avoid unnecessary travel and exposure during COVID-19, international cancer societies have recommended online video or telephonic consultation of cancer patients on follow-up [12]. In developing countries like India, web-based clinical assessment might not be a feasible alternative due to lack of resources and technology in the majority of patients.

There was considerable reduction in the number of new cases in both academic institutes (47.6%) and multi-specialty hospitals (70.8%) during COVID-19. The decrease in case load can be attributed primarily to intra- and inter-state transport restrictions and partly to fear of contracting COVID-19 among the public. The ominous delay in seeking medical attention during COVID-19 on the part of patients might lead to detrimental oncological outcomes in future. When compared to multi-specialty hospitals (29.2%), 61.9% of respondents in academic institutes found it difficult to maintain social distancing in OPD. This can be partly attributed to the large, heterogenous patient crowd in academic institutes compared to multi-specialty hospitals. Therefore, it is of prime importance to educate patients and attenders to maintain social distancing and follow safety precautions on hospital premises. Volunteers and hospital security personnel can be employed to monitor this. Work hours have been significantly lowered in respondents above 50 years (83.3%), which is a nice gesture from the hospital management to protect vulnerable elderly doctors from COVID-19. At the same time, the courage of respondents below 50 years to take on the responsibilities and serve cancer patients during these testing times should also be lauded.

It is quite evident from the responses that hospital management teams have taken adequate precautions to ensure the safety of healthcare workers as 42.7% respondents were satisfied with the safety measures. On the other hand, when it comes to the safety of patients undergoing radiotherapy, 43.9% respondents felt worried. This is an area to be addressed by every hospital. Allocation of specific radiation treatment timings for each patient, minimisation of pre-treatment waiting time, stringent rules on adherence to safety precautions such as wearing mask and social distancing must be imposed to improve patient’s safety. Despite 42.7% respondents feeling satisfied with safety measures in workplace, the fear of contracting COVID-19 infection (69.5%) and fear of infecting family members (78%) were high. Hospital managements should come up with solutions like conduct of weekly virtual counselling, yoga and meditation sessions to alleviate the fears and keep the morale of healthcare workers high during the ongoing COVID-19 pandemic. Improving safety measures in hospital and supplying adequate protective kits also play an important part.

Neither the interaction of radiation oncologists with physicists during treatment planning, nor their physical presence during new case implementation or daily setup image verification had reduced during COVID-19. Almost three-fourths of respondents were continuing their routine just as pre-COVID times. It is high time that radiation oncologists route to newer technologies to verify patient’s treatment position from their consultation room, through apps or server-based transfer of setup images, instead of spending more time in the treatment room. The adoption and utilisation of technology, be it for follow-up or radiotherapy image verification, has been minimal in India [15]. Radiotherapy to cancer patients was deferred by 35.7% of respondents from academic institutes and 29.2% from multi-specialty hospitals due to COVID-19 risk. Such decisions are to be taken on a case-by-case basis after carefully weighing the risks and benefits.

Radiation Oncology societies and academic institutes have recommended various hypofractionation and shorter radiation treatment regimens during COVID-19 [8–11]. There has been a constant urge to follow these recommendations during COVID-19 to avoid unwanted exposure and treatment prolongation. However, in our survey, only about half of the respondents (51.2%) switched to newer fractionation schedules, while the remaining half (49.8%) were still reluctant to adopt them. Among the respondents who adopted new schedules, few were keen to continue them even after the end of COVID-19 pandemic. The lack of interest to adopt new fractionation schedules might be due to the prevailing doubts over their effectiveness, and lack of validation. As leading oncology institutions begin to share their experiences through webinars and publications, the awareness and faith over these new fractionation schedules will improve. Few respondents showed interest in following the new radiotherapy schedules post-COVID-19 pandemic too. Studies have shown single fraction radiotherapy to be equally effective as multi-fraction radiotherapy for palliation of painful bone metastasis [16, 17]. Despite the encouraging study results and present COVID-19 situation, only less than half (42.7%) of the respondents adopted single fraction radiotherapy for pain palliation. Single fraction radiotherapy could be a cost-effective, resource-efficient and exposure-curbing alternative for pain palliation during COVID-19.

In addition to the challenges in treating cancer patients, the COVID-19 pandemic also poses a serious threat to cancer research and discoveries. Research works were reduced in 62.2% of the respondents. From a radiation oncology trainee standpoint, 75% of the post-graduates reported a decrease in cancer research during COVID-19. This can be attributed to reduction in volume of patients, physical examination and interaction time with senior doctors. The clinical skills and professional growth of the residents are hampered which might reflect cancer care in future. Finally, regarding job satisfaction, 41.5% of respondents were satisfied with the care provided to the cancer patients during COVID-19, while 30.5% were not satisfied.

Studies conducted in other developing countries such as Latin America and Turkey showed similar trends in reduction of new cancer cases and availability of personal protective equipment. The adoption of hypofractionation was less in Latin America like our study, whereas it was more in Turkey [18, 19]. None of these or similar studies focused on the impact of COVID-19 on the psychological state of radiation oncologists and cancer research.

The main strength of our study was its ability to capture real world information regarding the adoption of new radiotherapy schedules, and psychological state of radiation oncologists during COVID-19 pandemic. There were two limitations to this survey. First, although the hyperlink was unique, it was publicly available and there was no control over the possibility that the same person answered the survey multiple times. However, that does not seem to be the case seeing the diversity of responses. Secondly, COVID-19 infections are rapidly spreading throughout the country and globally. Hence, guidelines for cancer treatment and COVID-19 control measures are constantly evolving. This might render few of the responses to become invalid when the guidelines are modified depending on the COVID-19 situation.

## Conclusion

The COVID-19 pandemic has created a major change in the work life of radiation oncologists in India. Despite the fear of contracting COVID-19, radiation oncologists have continued to provide cancer care during this pandemic by resorting to newer norms. However, further incorporation of technology and safety precautions in daily practice is warranted.

## Funding

The authors have declared that no specific grant was obtained for this research from any funding agency in the public, commercial or not-for-profit sectors.

## Conflicts of interest

The authors have no conflict of interest to declare.

## Online Supplementary Material

**Life of Radiation Oncologists during COVID-19**

A short survey on how life as RO has changed

1. Gender *
  *Mark only one oval*.
  - Male
  - Female
  - Prefer not to say
2. Age *
  *Mark only one oval*.
  - 20-30 years
  - 31-40 years
  - 41-50 years
  - >51 years
3. Position *
  *Mark only one oval*.
  - Post Graduate
  - Senior Resident/Registar
  - Assistant professor/Consultant
  - Professor/Head of Department
4. Institution *
  *Mark only one oval*.
  - Cancer clinic
  - Academic institute
  - Multi speciality hospital
  - Government hospital
5. Screening programs & camps *
  *Mark only one oval*.
  - Conducted
  - Stopped for time being
  - Cancelled for the whole year
  - Not applicable
6. Follow-up *
  *Mark only one oval*.
  - In-person examination
  - Online consultation
  - Telephonic interview
  - Postponed for another 6 months
7. New cases *
  *Mark only one oval*.
  - Reduced
  - Increased
  - Same as pre-COVID time
8. Work timing *
  *Mark only one oval*.
  - Weekly/3-day shifts
  - Daily routine timings
  - Daily but reduced timings
9. Social distancing in OP *
  *Mark only one oval*.
  - Maintained
  - Difficult to maintain
  - Unable to maintain
10. Safety measures to healthcare workers *
  *Mark only one oval*.
  - Extremely satisfied
  - Satisfied
  - Neutral
  - Worried
  - Extremely worried
11. Safety of on-RT patients *
  *Mark only one oval*.
  - Extremely satisfied
  - Satisfied
  - Neutral
  - Worried
  - Extremely worried
12. Fear of contracting COVID *
  *Mark only one oval*.
  - Extremely high
  - High
  - Neutral
  - Low
  - Extremely low
13. Fear of infecting family members *
  *Mark only one oval*.
  - Extremely high
  - High
  - Neutral
  - Low
  - Extremely low
14. Interaction with physicist during plan evaluation *
  *Mark only one oval*.
  - Reduced
  - Increased
  - Same as pre-COVID
15. Physical presence during new case implementation *
  *Mark only one oval*.
  - Reduced
  - Increased
  - Same as pre-COVID time
16. Physical presence to check CBCT/setup images *
  *Mark only one oval*.
  - Reduced
  - Increased
  - Same as pre-COVID time
17. Deferred/Refused RT due to COVID-19 *
  *Mark only one oval*.
  - Yes
  - No
18. Adopted new fractionation schedules (hypofractionation) *
  *Mark only one oval*.
  - Yes
  - No
19. Mention sites where new fractionation schedules adopted
20. Would you like to continue the new schedules post COVID ? *
  *Mark only one oval*.
  - Yes
  - No
  - Maybe
  - Not applicable
21. Preference for single fraction palliative RT *
  *Mark only one oval*.
  - Increased
  - Reduced
  - Same as pre-COVID
22. Research work and publications *
  *Mark only one oval*.
  - Reduced
  - Increased
  - Same as covid time
23. How satisfied are you with your patient care during COVID 19 ? *
  *Mark only one oval*.
  - Extremely satisfied
  - Satisfied
  - Neutral
  - Not satisfied
  - Regretful

* Required
